# Patterns of alcohol and drug screening in trauma patients: understanding housing status as a determinant of care

**DOI:** 10.1186/s40621-026-00687-0

**Published:** 2026-05-12

**Authors:** John Beckerle, Vlada Stark, Bertille Assoumou

**Affiliations:** Kirk Kerkorian School of Medicine at UNLV, Las Vegas, NV USA

**Keywords:** Trauma, Social determinants of health, Homelessness, Drugs, Alcohol

## Abstract

**Background:**

Traumatic injury remains a leading cause of morbidity and mortality in the United States, with substance use and social determinants of health playing important roles in injury risk and clinical outcomes. People experiencing homelessness are disproportionately exposed to trauma and are screened for alcohol and drugs at higher rates than housed patients, raising questions about whether differences in substance positivity reflect true variation in exposure or disparities in screening practices. This study examines patterns of alcohol and drug screening and positivity among housed and unhoused trauma patients.

**Methods:**

We conducted a retrospective cross-sectional analysis of adult trauma patients included in the 2021 National Trauma Data Bank. Alcohol and drug screening within the first 24 h of hospital encounter were examined, along with screening results and the presence of multiple concurrent drug positives. Homelessness was the primary exposure of interest. Multivariable logistic regression models were used to assess the association between housing status and screening practices as well as positive alcohol, drug, and polydrug results, adjusting for age, sex, race, ethnicity, injury severity, Glasgow Coma Scale score, physical and behavioral comorbidities.

**Results:**

Among 1,000,269 adult trauma patients, 9,466 (0.9%) were unhoused. Unhoused patients were significantly more likely to be screened for alcohol and drugs than housed patients. After adjustment, homelessness remained the strongest predictor of screening for both alcohol and drugs. Among those screened, homelessness was associated with markedly higher odds of positive drug and polydrug results. The association with positive blood alcohol concentration was modest despite substantially higher screening rates. Injury severity and lower Glasgow Coma Scale scores were also associated with increased screening and positivity.

**Conclusions:**

Unhoused trauma patients experience substantially higher rates of alcohol and drug screening and higher odds of drug and polysubstance positivity compared with housed patients. These findings suggest that housing status strongly shapes diagnostic practices in trauma care and may influence the interpretation of substance use epidemiology. Standardized, non-stigmatizing screening approaches that are paired with appropriate clinical and social interventions are needed to ensure equitable trauma care.

**Supplementary Information:**

The online version contains supplementary material available at 10.1186/s40621-026-00687-0.

## Background

Traumatic injury remains a leading cause of morbidity and mortality in the United States (US), with recent decades marked by a sustained rise in trauma-related deaths driven in part by interpersonal violence and substance-related mechanisms [1]. Social determinants of health, particularly housing instability, play a critical role in shaping both trauma risk and clinical outcomes following injury. People experiencing homelessness are disproportionately exposed to environmental hazards, interpersonal violence, and untreated medical and psychiatric conditions, all of which increase the likelihood of traumatic injury and complicate recovery [2, 3]. As a result, trauma systems increasingly function as de facto safety nets for unhoused populations, often managing patients with complex medical and social needs that extend beyond the acute injury itself [2, 4]. There is evidence that acute drug intoxication and traumatic injury are large contributors to injury and mortality in the unhoused population [5].

Substance use is highly prevalent among people experiencing homelessness and represents an important, though methodologically complex, contributor to trauma risk. Epidemiologic studies have demonstrated elevated rates of alcohol and drug exposure in this population, with polysubstance use being common [4, 8–9, 11]. However, the clinical interpretation of substance use screening results in trauma settings is not straightforward. Prior work has shown that urine drug screen (UDS) positivity does not consistently predict perioperative complications or mortality and may reflect a combination of chronic use, acute intoxication, and medications administered after hospital admission [6]. These findings underscore the limitations of interpreting substance detection as a direct proxy for pre-injury substance use or trauma-related risk.

At the systems level, alcohol and drug screening practices in trauma care are highly variable. National analyses have demonstrated substantial institutional variation in screening adoption, with fewer than one-third of trauma patients screened overall [4, 7]. Importantly, people experiencing homelessness are screened more frequently than housed patients, which complicates interpretation of higher test positivity rates and raises concern for structural and institutional bias in diagnostic practices [4, 7–8]. Prior studies have also shown that unhoused trauma patients are more likely to be admitted to the hospital, experience longer lengths of stay, and utilize intensive care resources at higher rates, even after accounting for injury severity, highlighting the influence of social context and discharge safety concerns on clinical decision-making [4, 7–8].

Despite the well-documented overlap between homelessness, substance use, and traumatic injury, limited research has examined whether differences in substance positivity among unhoused trauma patients reflect true variation in pre-injury exposure or disparities in screening practices shaped by injury severity, clinician perception, and institutional norms. Existing studies have largely focused on single substances or opioid-related use, leaving an important gap in understanding polysubstance patterns and their relationship to housing status within trauma systems [9, 10]. Using data from the 2021 National Trauma Data Bank, the present study compares rates of alcohol and drug screening among housed and unhoused trauma patients to better characterize how housing status intersects with screening practices and substance use patterns in contemporary trauma care.

## Methods

### Study design and population

This retrospective cross-sectional study used data from the 2021 National Trauma Data Bank (NTDB), made available by the American College of Surgeons (ACS) and its Trauma Quality Programs (TQP) data. The ACS TQP includes data from participating hospitals in the US and Canada that adhere to the National Trauma Data Standard, which aims to set standards for clinical data elements that are important for trauma care. Patient demographic, clinical, injury, and hospital data are extracted without protected health information. The NTDB does not include data on costs, laboratory values, readmissions, or long-term outcomes [11]. The Institutional Review Board for the University of Nevada, Las Vegas determined that this study is exempt from the need for informed consent due to the deidentified nature of the dataset.

## Variables description

### Dependent variables

The outcomes we were interested in were the frequency of alcohol and drug screenings, screening results and the presence of multiple drugs detected concurrently, which we deemed polydrug use. The NTDB reports screening for alcohol and 13 types of drugs performed within the first 24 h of hospital presentation [11]. Screening status is recorded as a binary variable indicating whether a patient was screened for alcohol and/or drugs within their first 24 h of the hospital encounter. As such, the NTDB does not provide detailed information regarding the modality of drug testing (e.g., blood vs. urine), whether screening was protocolized, or the clinical rationale for testing. In general, alcohol screening reflects blood alcohol concentration (BAC) testing, while drug screening reflects urine or serum toxicology testing performed within the first 24 h of hospital presentation, although practices may vary across institutions.

Among screened patients, results are reported as a numeric ethanol level for alcohol in g/dL, and a binary result for each class of drugs. The 13 drug classes included in screening were opioids, amphetamine, barbiturates, benzodiazepine, cocaine, methamphetamine, ecstasy, methadone, oxycodone, phencyclidine, tricyclic antidepressants (excluded from this study), cannabinoids, and others [11]. For each patient, the number of positive drug classes was calculated and the presence of two or more positive drug classes was defined as polydrug use. A BAC ≥ 0.08 g/dL (0.08%) was considered positive for this study.

### Independent variables

Homelessness was the primary risk factor of interest for us to examine disparities in screening practices. Homelessness, as defined by the NTDB, refers to individuals lacking permanent housing, including those living in transitional housing or temporary living quarters. Homelessness is recorded as a patient-level variable by trained NTDB data abstractors using patient identification documents available at the time of care. In accordance with protected health information regulations, postal codes are not available to researchers. For individuals without a temporary or permanent residence ZIP code listed, clinical abstractors are trained to record these patients’ ZIP code as not applicable and complete the alternate home residence variable [8]. Patients with the alternate home residence variable for homelessness were considered unhoused for our data analysis.

### Covariates

Covariates included in multivariate modeling include age, sex, race, ethnicity, injury severity score (ISS), total Glasgow Coma Scale (GCS) upon admission, and presence of physical and behavioral comorbidities. These variables were collected for both housed and unhoused patients. The NTDB collects race and ethnicity data from self-report or report by family members and records race and ethnicity as separate variables. Categories included Hispanic and non-Hispanic for ethnicity, and Black, White, Asian, Pacific Islander and other for race. Patients in either the Asian or Pacific Islander category were combined into a single AAPI category to improve statistical stability and consistency with prior literature. Comorbidities are assigned by ICD-10 comorbidity codes (Supplementary Material 1). Physical comorbidities include conditions such as heart disease, diabetes, hypertension, liver disease, and malignant neoplasms. Behavioral comorbidities include bipolar disorder, major depressive disorder, posttraumatic stress disorder, and other major psychiatric conditions. Substance comorbidities include alcohol use disorder and substance use disorder, which encompasses use of stimulants, opioids and other drugs of abuse [11].

### Statistical analysis

Chi-square tests of independence were used to assess significant differences between housed and unhoused patients. Missing values for any demographic information were included in a descriptive statistics table for clarity. Univariate logistic regression models were used to estimate the unadjusted odds ratios for the association between homelessness on alcohol and drug screening, and the results of these screens. Multivariate logistic regression models were used to estimate adjusted odds ratios to more closely examine the relationship between homelessness and screening outcomes while controlling for potential confounders. Covariates included age, sex, race, ethnicity, ISS, GCS score on presentation, and selected physical, behavioral and substance comorbidities. We conducted multivariate analysis for five outcomes: alcohol screening, drugs screening, positive screenings for alcohol, drug and polydrug use. All statistical analyses were performed in R4.4.0. Statistical significance was set at *P* < 0.05.

For regression models, all continuous variables were categorized into discrete intervals: age intervals were 18–34, 35–49, 50–64, and 65 or older; GCS categories were 13–15, 9–12, and 3–8; ISS categories were mild (1–8), moderate (9–15), and severe (16 or higher).

## Results

### Descriptive analysis

The total sample size of the 2021 NTDB data set is 1,209,097 patients. After removing those under the age of 18 years or with a missing age, 1,000,269 patients were included in the present study. Of these, 9,466 (0.9%) were unhoused. In the housed cohort, 587,009 (59.2%) were male whereas 7,845 (82.9%) of the unhoused patients were male. Unhoused patients were less likely to be elderly (65 or older) (7.2% vs. 39.6%). Although White people made up the majority of the patient population in both groups, there was a higher prevalence of Hispanic and Black patients in the unhoused cohort (2,024 [21.4%] and 2,118 [22.4%] vs. 116,723 [11.8%] and 148,753 [15.0%]). Medicaid and self-pay were the most common forms of primary payment for unhoused patients (4,570 [48.3%] and 2,030 [21.3%]) while the majority of housed patients used either Medicare or private insurance (345,416 [34.9%] and 312,470 [31.5%] respectively).

Unhoused patients had higher instances of severe ISS (1,978 [20.9%] vs. 167,179 [16.9%], *p* < 0.001). They were also more likely to present with GCS scores in the moderate category (9–12: 801 [8.5%] vs. 55,549 [5.6%]), compared to housed patients. The presence of diagnosed physical, behavioral, or substance comorbidity was similar between both groups. Table 1 presents a summary of patient characteristics.

**Table 1:** Patient Characteristics by Housing Status in the 2021 National Trauma Data Bank Dataset

Patient Characteristics	Housed *n* (%)	Unhoused *n* (%)	*p* Value*
All	990,803 (99.1)	9,466 (0.9)	<0.001
Sex			<0.001
Male	587,009 (59.2)	7,845 (82.9)	
Female	394,977 (39.8)	1,533 (16.1)	
Missing	271 (0.0)	2 (0.0)	
Age (years)			<0.001
18-34	248,377 (25.1)	2,910 (30.7)	
35-49	163,305 (16.5)	3,110 (32.9)	
50-64	186,446 (18.8)	2,760 (29.2)	
65+	392,675 (39.6)	686 (7.2)	
Race			<0.001
White	715,233 (72.2)	5,506 (58.2)	
Black	148,753 (15.0)	2,118 (22.4)	
AAPI	23,652 (2.4)	206 (2.2)	
Other/Missing	103,165 (10.4)	1,636 (17.3)	
Ethnicity			<0.001
Hispanic	116,723 (11.8)	2,024 (21.4)	
Non-Hispanic	839,178 (84.7)	7,080 (74.8)	
Missing	34,902 (3.5)	362 (3.8)	
Insurance			<0.001
Medicaid	151,935 (15.3)	4,570 (48.3)	
Medicare	345,416 (34.9)	909 (9.6)	
Other Government	31,467 (3.2)	452 (4.7)	
Private/Commercial	312,470 (31.5)	1,256 (13.2)	
Self-Pay	104,952 (10.6)	2,030 (21.3)	
Other	22,215 (2.2)	143 (1.5)	
Not Billed (for any reason)	2,363 (0.2)	18 (0.2)	
Missing	19,985 (2.0)	88 (0.9)	
Comorbidity			
Physical	488,254 (49.3)	4,641 (49.0)	0.635
Behavioral	172,374 (17.4)	1,603 (16.9)	0.242
Substance	122,655 (12.4)	1,110 (11.7)	0.057
ISS			<0.001
Mild (1-8)	456,982 (46.1)	4,639 (49.0)	
Moderate (9-15)	364,460 (36.8)	2,925 (29.8)	
Severe (≥16)	167,179 (16.9)	1,978 (20.9)	
Missing	2,182 (0.2)	24 (0.3)	
GCS			<0.001
13-15	859,321 (86.7)	7,772 (82.1)	
9-12	21,606 (2.2)	533 (5.6)	
3-8	55,549 (5.6)	801 (8.5)	
Missing	54,327 (5.5)	360 (3.8)	

AAPI: Asian American or Pacific Islander

ISS: Injury Severity Score

GCS: Glasgow Coma Scale

*Determined by Chi-square tests of independence

Alcohol screening was conducted for 7,135 unhoused patients (75.4%) and 462,134 housed patients (46.6%) while drug screening was performed for 5,733 (60.6%) unhoused and 312,212 housed (31.5%) patients. Of those screened, 1,926 (27.0%) unhoused and 100,525 (21.8%) housed patients tested positive for alcohol, whereas 4,270 (74.4%) unhoused and 140,041 (44.9%) housed patients tested positive for drugs; polydrug use was detected in 2,424 (42.3%) and 54,435 (17.4%) patients, respectively.

### Univariate analysis

In univariate logistic regression analysis, unhoused patients had more than three times the odds of getting screened on admission for drugs and alcohol compared to housed patients (OR: 3.34, 95% CI: 3.20, 3.48 and OR: 3.50, 95% CI: 3.34, 3.67, respectively). Of those screened, the unhoused were around five times as likely to test positive for drug and polydrug use (OR: 4.99, 95% CI: 4.79, 5.20 and OR: 5.92, 95% CI: 5.65, 6.21), but moderately more likely to test positive for alcohol (OR: 1.37, 95% CI: 1.30, 1.44), despite being screened more.

### Multivariate analysis

When accounting for confounders, homelessness remained the strongest determinant of whether a patient was screened for substance use. This was true for both alcohol and drug screenings, with unhoused patients having more than twice the odds of being screened (aOR: 2.37, 95% CI: 2.25, 2.49 vs. aOR: 2.42, 95% CI: 2.31, 2.53, respectively). Black patients were consistently screened more than white patients for alcohol (aOR: 1.16, 95% CI: 1.14, 1.17) and drugs (aOR: 1.12, 95% CI: 1.10,1.13); Hispanic patients were more likely to be screened for alcohol (aOR 1.21, 95% CI: 1.19, 1.23 ) and drugs (aOR: 1.24, 95% CI: 1.22, 1.26) than non-Hispanics. Female trauma patients were less likely to be screened for alcohol (aOR: 0.68, 95% CI: 0.67, 0.69) and drugs (aOR: 0.76, 95% CI: 0.76, 0.77), compared to male patients.

Patients with moderate and severe ISS were screened more for alcohol, (aOR 1.17, 95% CI: 1.16, 1.18 and aOR: 2.03, 95% CI: 2.00, 2.06, respectively), but less for drugs (aOR: 0.90, 95% CI: 0.89, 0.92 and aOR 0.88, 95% CI: 0.86, 0.90, respectively). Patients with a GCS of 9–12 and 3–8 were screened more than patients with a GCS of 13–15 for both alcohol and drugs, respectively (aOR: 2.31, 95% CI: 2.23, 2.38 and aOR: 2.46, 95% CI: 2.39, 2.53 vs. aOR: 1.21, 95% CI: 1.18, 1.23 and aOR: 1.60, 95% CI: 1.57, 1.63). Increasing age was associated with progressively lower odds of substance use screening, with the lowest odds among the elderly for both alcohol and drug screenings (aOR: 0.36, 95% CI: 0.36, 0.37 and aOR: 0.43, 95% CI: 0.42, 0.43, respectively).

Patients with a known physical comorbidity were screened less for alcohol (aOR: 0.98, 95% CI: 0.98, 0.99) and more for drugs (aOR 1.06, 95% CI: 1.05, 1.07). Behavioral comorbidities were associated with decreased odds for alcohol screening (aOR 0.95, 95% CI 0.94, 0.97) and no significant change in drug screening. Substance comorbidities were associated with decreased chances of alcohol (aOR: 0.91, 95% CI: 0.90, 0.93) and drug screening (aOR: 0.92, 95% CI: 0.90, 0.94). (Table 2).

**Table 2:** Multivariate Analysis of Patient Characteristics Affecting Alcohol and Drug Screening in Trauma Patients

Patient Characteristics	Alcohol aOR (95% CI)	Drug aOR (95% CI)
Housing Status		
Housed Homelessness	[REF] 2.37 (2.25, 2.49)	[REF] 2.42 (2.31, 2.53)
Race		
White Black AAPI Other	[REF] 1.16 (1.14, 1.17) 1.44 (1.39, 1.47) 1.12 (1.09, 1.13)	[REF] 1.12 (1.10, 1.13) 0.89 (0.86, 0.91) 1.00 (0.98, 1.02)
Ethnicity		
Non-Hispanic Hispanic	[REF] 1.21 (1.19, 1.23)	[REF] 1.24 (1.22, 1.26)
Sex		
Male Female	[REF] 0.68 (0.67, 0.69)	[REF] 0.76 (0.76, 0.77)
ISS		
Mild (1-8) Moderate (9-15) Severe (≥16)	[REF] 1.17 (1.16, 1.18) 2.03 (2.00, 2.06)	[REF] 1.24 (1.23, 1.25) 1.88 (1.85, 1.90)
GCS		
13-15 9-12 3-8	[REF] 2.31 (2.24, 2.38) 1.21 (1.18, 1.23)	[REF] 2.46 (2.39, 2.53) 1.60 (1.57, 1.63)
Age		
18-34 35-49 50-64 65 and older	[REF] 0.90 (0.88, 0.91) 0.71 (0.70, 0.72) 0.36 (0.36, 0.37)	[REF] 0.97 (0.96, 0.98) 0.79 (0.78, 0.80) 0.43 (0.42, 0.43)
Comorbidity		
Physical Behavioral Substance	0.98 (0.98, 0.99) 0.95 (0.94, 0.97) 0.91 (0.90, 0.93)	1.06 (1.05, 1.07) 0.99 (0.98, 1.01) 0.94 (0.92, 0.96)

aOR: Adjusted Odds Ratio

CI: Confidence Interval

AAPI: Asian American or Pacific Islander

ISS: Injury Severity Score

GCS: Glasgow Coma Scale

Homelessness was associated with a three- and fourfold increase in the odds of positive drug and polydrug screening results respectively (aOR: 3.38, 95% CI: 3.23, 3.53 vs. 4.06, 95% CI: 3.86, 4.27). However, the effect of homelessness on positive alcohol screenings was much smaller, though still statistically significant (aOR 1.07, 95% CI: 1.01, 1.13). As compared to white race, Black race was associated with higher odds of positive tests for alcohol, drugs and polydrug use (aOR: 1.09, 95% CI: 1.07, 1.11; aOR: 1.35, 95% CI: 1.33, 1.37; and aOR: 1.12, 95% CI: 1.10, 1.15, respectively). Hispanic ethnicity was not significantly associated with alcohol or drug screening positivity but was associated with lower odds of polydrug use (aOR: 0.88, 95% CI: 0.86, 0.91). Female patients had lower odds of testing positive for alcohol and drugs (aOR: 0.73 95% CI: 0.72, 0.74 and aOR: 0.72, 95% CI: 0.71, 0.73) as well as polydrug use (aOR: 0.74 95% CI: 0.72, 0.75).

Higher ISS were associated with lower odds of positive alcohol screenings for both moderate and severe injuries (aOR: 0.90, 95% CI: 0.89, 0.92 and aOR: 0.88, 95% CI: 0.86, 0.90 respectively). Conversely, higher ISS were associated with higher odds of positive drug screenings (aOR: 1.33, 95% CI: 1.31, 1.35 and aOR: 1.67, 95% CI: 1.64, 1.70 respectively). This pattern was also observed for polydrug use (aOR: 1.34, 95% CI: 1.31, 1.37 and aOR: 1.51, 95% CI: 1.48, 1.55).

GCS scores of 3–8 had increased odds of positive alcohol test (aOR: 1.63, 95% CI: 1.58, 1.66) more so than for drug (aOR: 1.38, 95% CI: 1.35, 1.41) or polydrug (aOR: 1.26, 95% CI: 1.22, 1.30). GCS scores of 9–12 were also associated with higher odds of positive screens for drug (aOR: 2.11, 95% CI: 2.04, 2.18) and polydrug (aOR: 1.95, 95% CI: 1.86, 2.04) than alcohol (aOR: 1.70, 95% CI: 1.64, 1.76). Elderly patients (over age 65) had the lowest risk of a positive alcohol (aOR: 0.35, 95% CI: 0.35, 0.36), drug (aOR: 0.13, 95% CI: 0.13, 0.13), or polydrug screen (aOR: 0.09, 95% CI: 0.09, 0.09).

Patients with a known physical comorbidity had no significant change in odds of alcohol positivity. However, physical comorbidity was associated with an increase in drug (aOR: 1.06, 95% CI: 1.04, 1.07) and polydrug positivity (aOR: 1.06, 95% CI: 1.04, 1.09) A known behavioral comorbidity was associated with a decrease in odds of a positive BAC screen (aOR: 0.92, 95% CI: 0.89, 0.94). There were no significant correlations between behavioral comorbidity and drug or polydrug positivity. A known substance use disorder was associated with increased risk of positive BAC screen (aOR: 1.11, 95% CI: 1.08, 1.15). Substance comorbidity was associated with a decreased drug positivity (aOR: 0.96, 95% CI: 0.94, 0.99) but no association with polydrug positivity. (Table 3).

**Table 3:** Multivariate Analysis of Patient Characteristics Associated with Positive Alcohol and Drug Screening Results in Trauma Patients

Patient Characteristics	Alcohol aOR (95% CI)	Drug aOR (95% CI)	Polydrug aOR (95% CI)
Housing Status			
Housed Homelessness	[REF] 1.07 (1.01, 1.13)	[REF] 3.38 (3.23, 3.53)	[REF] 4.06 (3.86, 4.27)
Race			
White Black AAPI Other/Missing	[REF] 1.09 (1.07, 1.11) 0.57 (0.54, 0.61) 1.24 (1.21, 1.27)	[REF] 1.35 (1.33, 1.37) 0.50 (0.47, 0.52) 0.93 (0.91, 0.95)	[REF] 1.12 (1.10, 1.15) 0.49 (0.46, 0.53) 0.92 (0.89, 0.95)
Ethnicity			
Not Hispanic Hispanic	[REF] 1.01 (0.99, 1.04)	[REF] 0.99 (0.97, 1.01)	[REF] 0.88 (0.86, 0.91)
Sex			
Male Female	[REF] 0.73 (0.72, 0.74)	[REF] 0.72 (0.71, 0.73)	[REF] 0.74 (0.72, 0.75)
ISS			
Mild (1-8) Moderate (9-15) Severe (≥16)	[REF] 0.90 (0.89, 0.92) 0.88 (0.86, 0.90)	[REF] 1.33 (1.31, 1.35) 1.67 (1.64, 1.70)	[REF] 1.34 (1.31, 1.37) 1.51 (1.48, 1.55)
GCS			
13-15 9-12 3-8	[REF] 1.70 (1.64, 1.77) 1.62 (1.58, 1.66)	[REF] 2.11 (2.04, 2.18) 1.38 (1.35, 1.41)	[REF] 1.95 (1.86, 2.04) 1.26 (1.22, 1.30)
Age			
18-34 35-49 50-64 65 and older	[REF] 1.08 (1.06, 1.10) 0.95 (0.93, 0.97) 0.36 (0.35, 0.36)	[REF] 0.87 (0.86, 0.88) 0.54 (0.53, 0.55) 0.13 (0.13, 0.13)	[REF] 1.04 (1.02, 1.07) 0.55 (0.54, 0.56) 0.09 (0.09, 0.09)
Comorbidity			
Physical Behavioral Substance	1.01 (0.99,1.02) 0.92 (0.89, 0.94) 1.11 (1.08, 1.15)	1.06 (1.04, 1.07) 0.98 (0.96, 1.00) 0.96 (0.94, 0.99)	1.06 (1.04, 1.09) 0.97 (0.94, 1.01) 0.99 (0.96, 1.03)

aOR: Adjusted Odds Ratio

CI: Confidence Interval

AAPI: Asian American or Pacific Islander

ISS: Injury Severity Score

GCS: Glasgow Coma Scale

## Discussion

To our knowledge, this is the first NTDB study to examine factors contributing to disparities in substance use screening between housed and unhoused trauma patients. Consistent with other studies, there are notable disparities in alcohol and drug screening among housed and unhoused trauma patients [7, 12]. Homelessness and a GCS of 9–12 were the strongest determinants for whether a patient was screened for alcohol or drugs. Importantly, of those screened, homelessness was the single strongest predictor of drug and polydrug positivity. However, the association between homelessness and positive alcohol screening was modest, despite patients being screened for alcohol two-to-threefold more frequently. This contrast likely reflects differences in detection windows and clinical workflows: alcohol intoxication is transient and more uniformly screened in trauma settings, whereas drug screening captures longer-term exposure and may be influenced by selective testing practices. Consequently, this may show that alcohol use is more generalized among the trauma population than drug use.

Housing status influences both the patients’ injury risk and their hospital care. Consistent with other studies, we found that unhoused trauma patients presented with more severe injuries than housed patients [7–10]. Yet injury severity alone did not fully explain the disparities in screening practices. Higher ISS was associated with more alcohol screening and less UDS, likely reflecting practical differences in screening modalities. Screening modalities were also influenced by demographic factors. Compared to housed patients, unhoused patients were more often younger, male, Black, and/or Hispanic, characteristics associated with higher screening rates. Despite being screened more, many of these demographic characteristics did not alter rates of positive screenings. Hispanic patients, in particular, did not test positive for any substance more than other groups, and had lower odds of polydrug positivity.

Our findings support broader epidemiologic evidence of high rates of polydrug exposure among people experiencing homelessness [4]. Prior work has shown that urine drug screening may reflect medications administered after hospital admission and thus may not always indicate pre-injury substance use [6]. Polydrug positivity may therefore function as a more stable epidemiologic marker of pre-injury substance exposure than single-substance results, which can be contaminated by in-hospital medications. In trauma datasets where toxicology timing is imprecise, prioritizing polydrug patterns may improve interpretability when examining substance-related injury risk across socially marginalized populations.

These findings also suggest that social context, clinician perception, and institutional norms play an important role in shaping diagnostic practices. The disproportionate screening of unhoused patients likely reflects a multifactorial relationship among provider perception, institutional norms, and visible social determinants of health. Such selective testing may reinforce stigma and the assumption that substance use is ubiquitous among unhoused individuals. Homelessness magnifies structural barriers in care, including longer hospital stays and delayed discharges, which may heighten clinician awareness and contribute to higher testing rates [6]. Even after adjusting for demographics and injury characteristics, housing status remained a strong independent predictor of alcohol and drug screening, highlighting the potential role of stigma in driving disparities in alcohol screening and positivity.

Selective screening carries implications for trauma care. Conflating visible social vulnerability with presumed substance use may influence clinical documentation, risk stratification, and discharge planning in ways that extend beyond toxicology itself, potentially shaping downstream care and patient–provider interactions. Without standardized, injury-based screening criteria, over screening of unhoused patients may distort substance-use epidemiology and reinforce stigmatizing perceptions.

The findings highlight the need for standardized, injury-based criteria for substance screening in trauma settings rather than reliance on social heuristics. Additionally, screening should be paired with appropriate interventions such as brief counseling, social work engagement, or harm-reduction referral. Trauma systems increasingly function as safety nets for socially marginalized populations, and equitable care requires that screening approaches minimize bias while ensuring that substance use identification is paired with appropriate intervention rather than punitive and stigmatizing responses [1, 7–9].

This study is subject to several limitations, including institutional variability, test timing after admission, and the binary housing variable, which may underestimate unstable living conditions. The institutions included in the NTDB dataset primarily represent level I and II trauma centers and may not be representative of all trauma centers [13]. There is the possibility of testing bias because most trauma patients are not receiving substance screening. Additionally, UDS may detect medications administered after hospital admission, particularly opioids used for acute pain management. Despite these limitations, this large, multicenter dataset provides valuable insight into social disparities in trauma-related substance use screening.

## Conclusion

Unhoused trauma patients were more likely to be screened and to test positive for alcohol, drugs, and polydrug use compared with housed patients. While unhoused trauma patients were screened at higher rates, differences in alcohol positivity were modest compared with marked disparities in drug and polydrug detection. Disparities in screening and positivity reflect both social determinants of health and institutional practices. Standardized, equitable screening approaches are necessary to minimize bias, accurately capture substance-use patterns, and ensure that identification is paired with meaningful clinical and social support rather than reinforcing stigma.

## Electronic Supplementary Material

Supplementary Material 1

## Data Availability

The data that support the findings of this study are available from the American College of Surgeons, but restrictions apply to the availability of these data, which was used under license for this study, and so are not publicly available. Data are however available from the authors upon reasonable request and with permission of the American College of Surgeons.

## Abbreviations

US: United States
ACS: American College of Surgeons
NTDB: National Trauma Data Bank
TQP: Trauma Quality Programs
AAPI: Asian and Pacific Islander
ISS: Injury Severity Score
GCS: Glascow Coma Scale
BAC: Blood Alcohol Concentration
UDS: Urine Drug Screening
aOR: Adjusted Odds Ratio
CI: Confidence Interval

## References

[1] Rhee P, Holcomb JB, Zangbar B. Evolving Epidemiology of Increasing Trauma Deaths in the United States (2000–2020). Ann Surg. 2025;281(6):976–81. 10.1097/SLA.0000000000006668. 39931816 10.1097/SLA.0000000000006668

[2] Miller JP, O’ Reilly GM, Mackelprang JL, Mitra B. Trauma in adults experiencing homelessness. Injury. 2020;51(4):897–905. 10.1016/j.injury.2020.02.086. 32147144 10.1016/j.injury.2020.02.086

[3] US Department of Housing and Urban Development. The 2024 Annual Homelessness Assessment Report (AHAR) to Congress. Published online December 2024. Accessed March 2. 2025. https://www.huduser.gov/portal/sites/default/files/pdf/2024-AHAR-Part-1.pdf

[4] Schaffer KB, Wang J, Nasrallah FS, et al. Disparities in triage and management of the homeless and the elderly trauma patient. Injury Epidemiol. 2020;7(1):39. 10.1186/s40621-020-00262-1. 10.1186/s40621-020-00262-1 PMC7358191 32654664

[5] Cawley CL, Kanzaria HK, Kushel M, Raven MC, Zevin B. Mortality Among People Experiencing Homelessness in San Francisco 2016–2018. J Gen Intern Med. 2022;37(4):990–1. 10.1007/s11606-021-06769-7. 33835316 10.1007/s11606-021-06769-7 PMC8904687

[6] Culhane JT, Freeman CA. The Effect of Illegal Drug Screening Results and Chronic Drug Use on Perioperative Complications in Trauma. J Emerg Trauma Shock. 2020 Oct-Dec;13(4):279–85. doi: 10.4103/JETS.JETS_141_19. Epub 2020 Dec 7. P MID: 33897145; PMCID: PMC8047956. 10.4103/JETS.JETS_141_19 PMC8047956 33897145

[7] Silver CM, Thomas AC, Reddy S, et al. Morbidity and Length of Stay After Injury Among People Experiencing Homelessness in North America. JAMA Netw Open. 2024;7(2):e240795. 10.1001/jamanetworkopen.2024.0795. 38416488 10.1001/jamanetworkopen.2024.0795 PMC10902734

[8] Silver CM, Thomas AC, Reddy S, et al. Injury patterns and hospital admission after trauma among people experiencing homelessness. JAMA Netw Open. 2023;6(6):e2320862. 10.1001/jamanetworkopen.2023.20862. 37382955 10.1001/jamanetworkopen.2023.20862 PMC10311388

[9] Khadka S, Bardes JM, Al-Mamun MA. Opioid-related polysubstance use and its effect on mortality and health resource utilization among trauma patients. Inj Epidemiol. 2023;10(1):54. 10.1186/s40621-023-00459-0 . PMID: 37872616; PMCID: PMC10594664. 37872616 10.1186/s40621-023-00459-0 PMC10594664

[10] Rook JM, Spurrier RG, Russell CJ, et al. Disparities in Screening for Substance Use Among Injured Adolescents. JAMA Netw Open. 2024;7(10):e2436371–2436371. 10.1001/jamanetworkopen.2024.36371. 39365586 10.1001/jamanetworkopen.2024.36371 PMC11452809

[11] American College of Surgeons: National Trauma Data Standard (NTDS). Accessed December 2. 2024. https://www.tn.gov/content/dam/tn/health/events/Natl%20Trauma%20Data%20Standard%20Data%20Dictionary%202021.pdf

[12] Elkbuli A, Dowd B, Flores R, Boneva D, Hai S, Mckenney M. Alcohol and Drug Testing in the National Trauma Data Bank: Does it Matter? Journal of Emergencies, Trauma, and Shock 12(2):p 97, Apr–Jun 2019. | 10.4103/JETS.JETS_106_18 10.4103/JETS.JETS_106_18 PMC6557060 31198274

[13] Hashmi ZG, Kaji AH, Nathens AB. JAMA Surg. 2018;153(9):852–3. 10.1001/jamasurg.2018.0483. Practical Guide to Surgical Data Sets: National Trauma Data Bank (NTDB). 10.1001/jamasurg.2018.0483 29617536

