# Supplementary material for: Patterns of alcohol and drug screening in trauma patients: understanding housing status as a determinant of care
Source: Inj Epidemiol. 2026 May 12;13:48. doi: 10.1186/s40621-026-00687-0 (PMC13248402; doi:10.1186/s40621-026-00687-0)
Supplement: Supplementary file 1 — Supplementary Material 1 [file 40621_2026_687_MOESM1_ESM.docx]

Supplemental Figure:

**Table S1.** NTDB Pre-Existing Conditions and Associated ICD-10-CM Codes

Alcohol Use Disorder – F10.20

Bleeding Disorder – D69.9

Currently Receiving Chemotherapy for Cancer – Z51.11

Congenital Anomalies – Q89.9

Congestive Heart Failure – I50.9

Current Smoker – F17.200

Chronic Renal Failure – N18.9

History of Cerebrovascular Accident – Z86.73

Diabetes Mellitus – E11.9

Disseminated Cancer – C79.9

Advanced Directive Limiting Care – Z66

Functionally Dependent Health Status – Z74.1

History of Angina – Z86.79

History of Myocardial Infarction – I25.2

History of Peripheral Vascular Disease – Z86.79

Hypertension – I10

Prematurity – P07.30

Chronic Obstructive Pulmonary Disease – J44.9

Steroid Use – Z79.52

Cirrhosis – K74.60

Dementia – F03.90

Major Psychiatric Illness – F99

Drug Use Disorder – F19.20

Attention Deficit Hyperactivity Disorder – F90.9

Anticoagulant Therapy – Z79.01

Angina Pectoris – I20.9

Mental/Personality Disorder – F99

History of Myocardial Infarction – I25.2

Peripheral Arterial Disease – I73.9

Substance Use Disorder – F19.20

Prematurity – P07.30

Pregnancy – Z33.1
